# Complete mitochondrial genome of *Ladislavia taczanowskii* (Cypriniformes: Gobionidae)

**DOI:** 10.1080/23802359.2021.1944385

**Published:** 2021-08-01

**Authors:** Qi An, Cuizhang Fu

**Affiliations:** Ministry of Education Key Laboratory for Biodiversity Science and Ecological Engineering, Coastal Ecosystems Research Station of the Yangtze River Estuary, Institute of Biodiversity Science and Institute of Eco-Chongming, School of Life Sciences, Fudan University, Shanghai, China

**Keywords:** Cypriniformes, Gobionidae, Ladislavia, Gobioninae

## Abstract

Mitochondrial genomes of two individuals of Tachanovsky’s gudgeon *Ladislavia taczanowskii* have been determined on the basis of Sanger dideoxy sequencing. The gene compositions of two genomes contain 13 protein-coding genes, 2 rRNA genes, 22 tRNA genes, and 1 control region with the same length 16,614 bp. The phylogenetic tree reveals that the monotypic genus *Ladislavia* is a sister group of the subfamily Gobioninae within the family Gobionidae.

The monotypic genus *Ladislavia* is represented by the single species Tachanovsky’s gudgeon, *Ladislavia taczanowskii* Dybowski 1869 that it is placed into the family Gobionidae within the order Cypriniformes (Tan and Armbruster 2018). *Ladislavia taczanowskii* is a small stream fish, distributed in the China, Korea, Mongolia, and Russia (Fricke et al. 2021). Two new mitochondrial genomes of *Ladislavia taczanowskii* were obtained in the present study. These data could be used to clarify – the systematic position of this species within the Gobionidae.

One of two *Ladislavia taczanowskii* individuals was collected from the Yichun (47°44′24″, 128°54′36″), Heilongjiang province, China, and another from the Kuandian, (40°42′00″, 124°44′24″), Liaoning province, China. The two specimens and DNA have been deposited in the Zoological Museum of Fudan University, China (Cuizhang Fu, czfu@fudan.edu.cn) with voucher FDZM-LTYChun20170829-03 and FDZM-LTKD 20161006-09. The methods for the genomic DNA extraction, thermocycling parameters of the polymerase chain reaction (PCR) and Sanger dideoxy sequencing in the present study, referred to Chai and Fu, 2020. The PCR amplifications included 10 pairs of primers (Primer1 to Primer3; Primer5; Primer7 to Primer9; Primer11 to Primer13) adopted from Chai and Fu, 2020, and three pairs of primers designed – during this study as follows: Primer4, LT-IleF 5′-GGACCACTTTGATAGAG-3′ and LT-COIR 5′-CCAAATACRAGATARAGGGT-3′; Primer6, LT-ATP8F 5′-ACTAGAGGTGGTCGGKAGTCA-3′ and LT-ATP6R 5′-GCTTGGTGTGCCATTARACGTTTTCTTG-3′; Primer10, LT-SerF 5′-ACYCACCRAGGAAGGACA-3′, and LT-ND5R 5′-CCTATTTTTCGGATGTCTTG-3′. New mitochondrial genomes were assembled based on contiguous and overlapping segments. Their annotations used the mitochondrial genome of *Pseudorasbora elongate* (KF245485; Chen et al. 2015) as reference genome. The ModelFinder (Kalyaanamoorthy et al. 2017) was used to select the best substitution models of five partitions (two rRNA genes together, overall tRNA genes, and each codon of protein-coding genes) based on the AIC criterion. IQ-TREE 1.6.2 (Nguyen et al. 2015) was used to reconstruct the phylogenetic relationships based on the maximum likelihood (ML) analysis with 1000 ultrafast bootstraps (UFBoot; Hoang et al. 2018).

The gene compositions of two new mitochondrial genomes (GenBank Accession No. MT897994 and MT897995) are the same to contain 13 protein-coding genes, 2 rRNA genes, 22 tRNA genes, and 1 control region with the same length 16,614 bp and similar A + T base composition of 55.27% or 55.28%. The two mitochondrial genomes also display the same patterns in codon use and gene arrangements. There are two kinds of start codons (ATG and GTG) and four types of stop codons (TAA, TA–, TAG, and T—) in the 13 protein-coding genes. The order of gene arrangements is the same as other published mitochondrial genomes of the family Gobionidae (e.g. Tong and Fu 2019; Yi and Fu 2020; Chai and Fu 2020). There are a total of 52 bp nucleotide difference between the two mitochondrial genomes. The phylogenetic tree (Figure 1) reveals that the monotypic genus *Ladislavia* is a monophyletic group, and it is a sister group of the subfamily Gobioninae within the family Gobionidae with relatively high Bootstrap confidence (85%).

**Figure 1.**
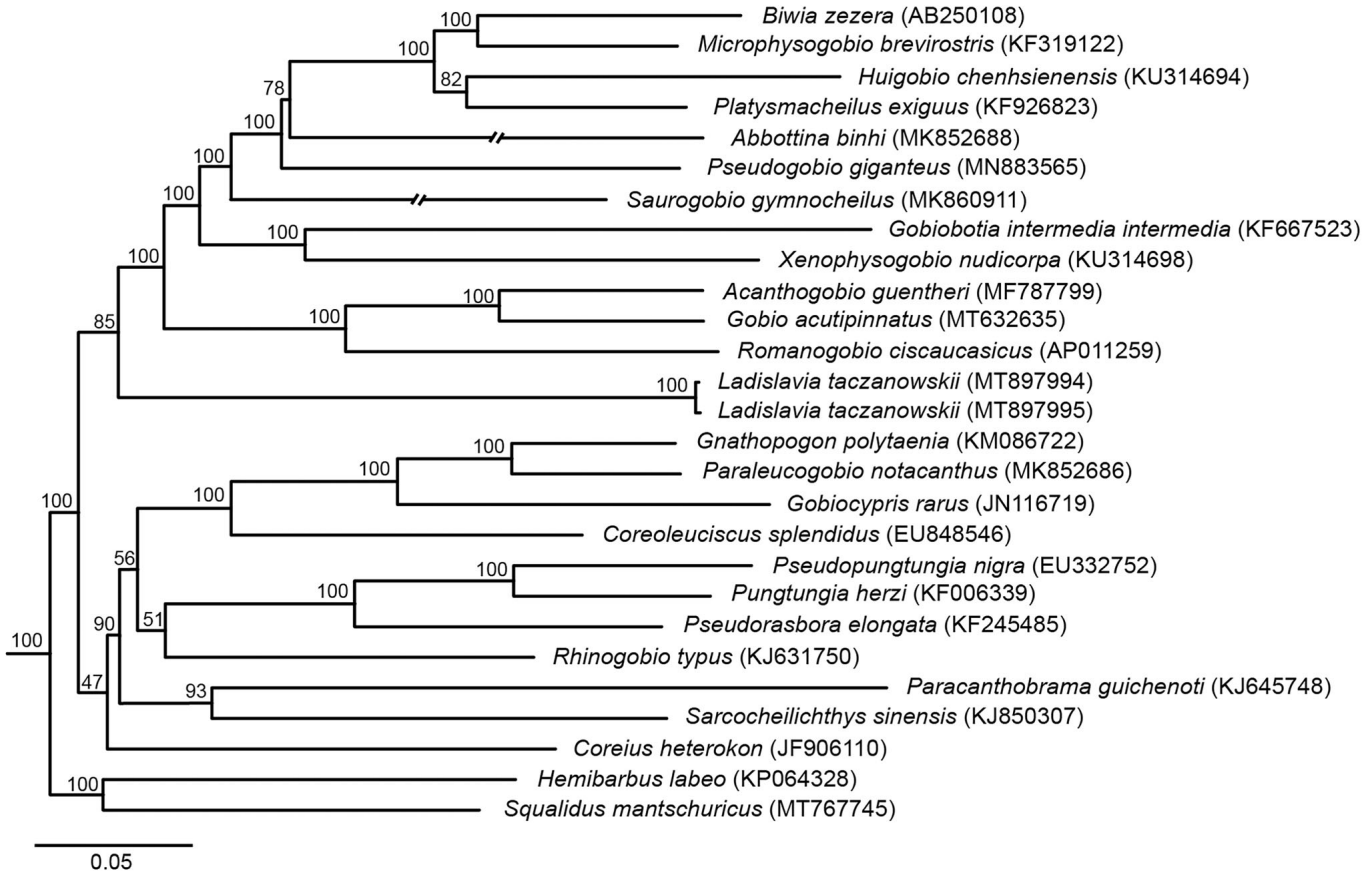
Phylogenetic relationships among *Ladislavia taczanowskii* and it close relatives within the family Gobionidae under a maximum likelihood analysis. The GenBank numbers are placed into in the parentheses and bootstrap confidences are shown on the above branches.

## Data Availability

Two new mitochondrial genomes with accession numbers MT897994 and MT897995 could be available in the GenBank: https://www.ncbi.nlm.nih.gov/nuccore/ MT897994 or MT897995.
